# *QPCTL* Affects the Daily Weight Gain of the F2 Population and Regulates Myogenic Cell Proliferation and Differentiation in Chickens

**DOI:** 10.3390/ani12243535

**Published:** 2022-12-14

**Authors:** Tuanhui Ren, Wujian Lin, Xiuxian Yang, Zihao Zhang, Shizi He, Wangyu Li, Zhuanjian Li, Xiquan Zhang

**Affiliations:** 1 Department of Animal Genetics, Breeding and Reproduction, College of Animal Science, South China Agricultural University, Guangzhou 510642, China; 2 Guangdong Provincial Key Lab of Agro-Animal Genomics and Molecular Breeding, and Key Laboratory of Chicken Genetics Breeding and Reproduction, Ministry of Agriculture, Guangzhou 510642, China; 3 College of Animal Science and Technology, Henan Agricultural University, Zhengzhou 450046, China

**Keywords:** *QPCTL*, Indel, daily weight gain, primary myoblast, cell proliferation

## Abstract

**Simple Summary:**

Molecular breeding can accelerate the process of animal breeding and improve breeding efficiency. At present, many Indel molecular markers have been identified in livestock and poultry, but how Indels affect economic traits is not well understood. For molecular breeding, it is crucial to reveal the mechanism of action of Indels and to provide more accurate information. The purpose of this study was to investigate how the 52/224-bp multiallelic Indels of the chicken *QPCTL* promoter area affect the daily weight gain of chickens and the potential regulatory mechanism of the *QPCTL* gene. The analysis was conducted by association analysis, qPCR, dual-fluorescence assay and Western blotting. The results of this study suggest that Indels in the promoter region of the *QPCTL* gene may regulate the proliferation and differentiation of chicken primary myoblasts by affecting the expression of *QPCTL*, which ultimately affects the growth rate of chickens. These Indels have important value for the molecular breeding of chickens, and *QPCTL* can be used as a candidate gene to regulate and improve chicken growth and development.

**Abstract:**

Molecular breeding can accelerate the process of animal breeding and improve the breeding efficiency. To date, many Indel molecular markers have been identified in livestock and poultry, but how Indels affect economic traits is not well understood. For molecular breeding, it is crucial to reveal the mechanism of action of Indels and to provide more accurate information. The purpose of this study was to investigate how the 52/224-bp multiallelic Indels of the chicken *QPCTL* promoter area affect the daily weight gain of chickens and the potential regulatory mechanism of the *QPCTL* gene. The analysis was conducted by association analysis, qPCR, dual-fluorescence assay and Western blotting. The results showed that Indels in the *QPCTL* promoter region were significantly associated with the daily weight gain in chickens and that *QPCTL* expression showed a decreasing trend in embryonic breast muscle tissues. Furthermore, *QPCTL* expression was significantly higher in breast muscle tissues of the AC genotype than in those of the AB and BB genotypes. Based on the transcriptional activity results, the pGL3-C vector produced more luciferase activity than pGL3-A and pGL3-B. In addition, overexpression of *QPCTL* promoted chicken primary myoblast (CPM) proliferation and inhibited differentiation. The results of this study suggest that Indels in the promoter region of the *QPCTL* gene may regulate the proliferation and differentiation of CPMs by affecting the expression of *QPCTL*, which ultimately affects the growth rate of chickens. These Indels have important value for the molecular breeding of chickens, and *QPCTL* can be used as a candidate gene to regulate and improve chicken growth and development.

## 1. Introduction

Chickens have a higher feed conversion rate than pigs and cattle, and chicken meat is China’s second most popular meat product after pork [1]. Therefore, the selection of chickens plays a very important role in animal husbandry. Animal growth characteristics are the most important economic traits, and the average daily weight gain (ADG) is an important target trait in animal breeding plans [2]. Muscle is a vital part of the animal body, and skeletal muscle accounts for approximately 40% of the body weight of animals [3]. Therefore, studying the muscle development of chickens is very important for livestock and poultry production.

Glutaminyl peptide cyclotransferase-like (*QPCTL*) is an intracellular enzyme that can mediate the N-terminal modification of a variety of substrates, which affects biological characteristics including monocyte chemoattractant protein-1 (*CCL2*) and monocyte chemoattractant protein-3 (*CCL7*), to protect them from proteolytic inactivation [4,5]. Previous studies in humans have shown that *QPCTL* is significantly related to body mass index [6]. *QPCTL* is evolutionarily conserved in chickens, zebrafish, humans and other animals and has important drug targeting characteristics [6,7]. In our previous study, seventeen chicken growth traits (including body weight at five different weeks of age and body length and other traits at two different weeks of age) and fourteen carcass traits (including evisceration, semi-evisceration, breast muscle and leg muscle weight, and other traits) were significantly correlated with the 52/224-bp multiallelic Indels in the chicken *QPCTL* gene promoter region [8]. However, because the functional mechanism of chicken *QPCTL* remains unclear, further exploration is necessary.

Molecular breeding can accelerate the process of animal breeding and improve the breeding efficiency. Gene mutations include SNPs and insertions/deletions (Indels), which are widely distributed in animal genomes and have been identified in large numbers in human and livestock research [9,10]. Indel mutations play an important role in research on the economic traits of domestic animals. A study showed a 10 bp Indel in the promoter region of cattle *PAX7*, and the deletion genotype increased the promoter activity and expression of *PAX7*, which then affected growth traits, such as the early weight and body length of cattle [11]. The 11 bp Indel of *DNMT3B* was significantly correlated with the first-born litter size of goats [12]. The 13 bp Indel mutation of pig *DGAT2* may affect fat deposition by modulating its mRNA expression [13]. A study of poultry found that a multiallelic Indel of *CDKN3* was significantly related to the carcass and growth traits of chickens [14]. A 22 bp Indel in the exon of the *ZNF764L* gene is significantly related to the body length, birth weight, chest circumference and other traits of chickens [15]. A 61 bp Indel in chicken *RIN2* changes the transcriptional activity of *RIN2* by affecting the transcription factor-binding site. This gene variation may affect fat deposition in chickens by influencing *RIN2* gene expression [16]. To date, few studies have examined how Indels affect animal economic traits. Further discovery of the mechanism of action of Indels will provide more accurate information in the field of molecular breeding.

The purpose of this study was to investigate if and how the 52/224-bp multiallelic Indels of the chicken *QPCTL* promoter area affect the daily weight gain of chickens, and the effects of *QPCTL* on the proliferation and differentiation of myoblasts (CPMs). We analyzed the correlation between Indels of the *QPCTL* gene and ADG of chickens, analyzed the expression of *QPCTL* in breast muscle tissues at different embryonic development stages and tissue expression profiles at different growth stages, and measured the expression changes of different *QPCTL* genotypes in tissues. In addition, the effect of Indels on the transcriptional activity of *QPCTL* was measured using dual-luciferase reporter assays, and a gene overexpression experiment was conducted to explore the function of *QPCTL* in CPMs. We found that 52/224-bp multiallelic Indels of the *QPCTL* gene were significantly related to the ADG of chickens. In particular, Indels can regulate the gene expression of *QPCTL*, and thus regulate the proliferation and differentiation of CPMs. In this study, we explored the role of the *QPCTL* gene in chicken growth and development and provided useful information for chicken molecular breeding.

## 2. Materials and Methods

### 2.1. Animal Samples and Trait Measurement

At 84 days of age, we collected blood samples from 804 chickens in a Gushi x Anka F2 resource population (F2 population) (373 males and 431 females), and genomic DNA was extracted with phenol-chloroform. The Gushi chicken (GS) is a slow-growing local Chinese breed, and the Anka chicken is a fast-growing broiler. All individuals had growth trait data records, and the methods used for measurement have also been described previously [10]. We used weekly weight measurement data to conduct a statistical analysis of the ADG of the F2 group at 0–4 weeks, 4–8 weeks, and 8–12 weeks.

A total of nine tissues were obtained from Gushi female chickens at 6, 14 and 75 weeks of age (leg muscle, breast muscle, abdominal fat, heart, liver, lung, kidney, sebum cutaneum and hypothalamus). *QPCTL* gene expression in the breast muscle of Xinghua chickens at six different embryonic periods (E10-15) was analyzed. Moreover, we collected breast muscle tissue from chickens fed high-protein diets and high-fat diets.

### 2.2. cDNA Synthesis and qRT-PCR

We extracted total RNA from tissue samples and cultured cells using a HiPure Universal RNA Kit (Magen, Guangzhou, China), and a cDNA reverse transcription kit (Vazyme, Nanjing, China) was used for reverse transcription. qRT-PCR was performed using the Universal SYBR qPCR Master Mix (Vazyme) and QuantStudio 5 PCR System (Applied Biosystems Inc., Foster, Waltham, MA, USA). We calculated the relative expression levels using the 2^–ΔΔCt^ method and determined their significance using Student’s t-test. Three or four biological and three technical repetitions were used for all reactions. The primers for the target genes and the internal control β-actin are presented in Supplementary Table S1.

### 2.3. Bioinformatics Analysis

MEGA 6.0 software was used to analyze the amino acid sequences of *QPCTL*s from twelve species. Before analysis, low consistency sequences were removed to achieve a stable topological structure.

The phylogenetic tree uses the Poisson correction model and neighbor-joining method. The *QPCTL* interacting protein of chicken was predicted using the online website String (https://cn.string-db.org/cgi/network?taskId=bZBM2EXClv9t&sessionId=bPpsyQ0kzZ8C, accessed on 25 November 2021). The transcription factor binding sites (TFs) in the 52/224-bp multiallelic Indels of the *QPCTL* gene were predicted using online Alibaba software (http://gene-regulation.com/pub/programs/alibaba2/index.html, accessed on 12 February 2022) [17].

### 2.4. Association Analysis

In our previous research, we found 52/224-bp multiallelic Indels in the promoter region of *QPCTL*, which is the same as the analysis model described in our previous article [8]. We used the method of Model I (Y_ijkl_ = µ + Gi + Sj + Hk + f_l_ + e_ijkl_) to analyze the correlation between different genotypes and the ADG of chickens at different growth stages. Y_ijkl_ represents the observed value, µ is the overall population mean, G_i_ is the fixed effect of genotype, S_j_ represents the fixed effect of sex, H_k_ is the fixed effect of hatch, f_l_ is the fixed effect of family, and e_ijkl_ is the random error.

### 2.5. Genotype Frequencies of QPCTL

To explore the proportion of six genotypes of *QPCTL* in different populations, we genotyped *QPCTL* by PCR. In addition, we used the published *QPCTL* genotype data and the newly added *QPCTL* genotype data to divide a total of 1570 chickens into three types, including dual-purpose type (Lushi, Changshun, Gushi, Xichuan, A line, D line, H line, M line, 103 line dwarf chicken, Xinghua dwarf chicken and Yellow dwarf chicken), white feathered broilers (Recessive White Rock chicken and Arbor Acres chicken), and yellow feathered broilers (Ma-Huang chicken) [8]. Statistical analyses were performed using GraphPad Prism software (version 8, San Diego, CA, USA).

### 2.6. Diets with Different Levels of Protein and Fat

The breast muscle tissue samples used to detect the expression of the *QPCTL* gene were provided by the Henan Innovative Engineering Research Center of Poultry Germplasm Resource. One-day-old chicks were randomly divided into three groups of diets with different protein levels, including the control group (20%), the low protein group (10%) and the high protein group (30%), 30 chickens were included in each group. The whole trial period was 21 days. Ten-week-old hens were randomly divided into a control group and a high-fat group (4% soybean oil), with 12 hens in each group, and the whole trial period was 30 days [18].

### 2.7. Cell Culture and Transfection

The DF1 cell line was obtained from the Key Laboratory of Chicken Genetics Breeding and Reproduction (Guangzhou, China). DF1 cells were cultured in basal DMEM (BI, Israel) containing 10% FBS (BI, Israel) and 1% streptomycin/penicillin (Invitrogen, Carlsbad, CA, USA) in an incubator containing 5% CO_2_ at 37 °C.

As previously mentioned, chicken primary myoblasts (CPMs) were isolated from the leg muscles of 11-day-old chicken embryos (E11) [19]. The obtained CPMs were cultured in RPMI-1640 medium (Gibco) supplemented with 1% penicillin/streptomycin and 20% FBS. In the differentiation experiment of CPMs, when CPMs reached 100% cell fusion, the growth medium was removed, and the cells were then cultured with differentiation medium (RPMI-1640, 2% horse serum and 0.1% penicillin/streptomycin). CPMs were incubated at 37 °C in an atmosphere with 5% CO_2_. All transient transfections were conducted with Lipofectamine 3000 reagent (Invitrogen).

### 2.8. Flow Cytometry Assays

To analyze the cell cycle of CPMs by flow cytometry, CPMs were inoculated into 12-well plates. When the CPMs grew to 50% confluence, they were transfected with the overexpression plasmid. Forty-eight hours after transfection, the CPMs were digested by trypsin, collected, fixed in 70% ethanol and stored at 4 °C. FxCycle™ PI/RNase staining solution (Invitrogen) was used to stain the fixed cells. After 30 min of staining at room temperature while shielding from light, a flow cytometry analysis was conducted using a BD Accuri^TM^ C6 flow cytometer (BD Biosciences, San Jose, CA, USA).

### 2.9. Dual-Fluorescence Detection

According to the site sequence diagram of Indels in the chicken *QPCTL* promoter region [8], the luciferase reporter vector pGL3-basic (Promega, Madison, WI, USA) was selected, and the pGL3-A allele vector (388 bp, including insertions of 224 and 52 bp), pGL3-B allele vector (336 bp, including a deletion of 52 bp and an insertion of 224 bp) and pGL3-C allele vector (164 bp, including an insertion of 52 bp) were constructed through Gene Create (Wuhan, China) to detect the effects of different Indels in the *QPCTL* promoter region on fluorescence activity. The above plasmids were transfected into DF-1 cells, and the TK Renilla reporter plasmid (Promega) was co-transfected as an internal control.

The luciferase activity of the cells was analyzed by a Luciferase Assay System Kit (Promega) and Fluorescence/Multi-Detection Microplate Reader (BioTek, Winooski, VT, USA) with Gen5 software. The levels of firefly luciferase activities were normalized to those of Renilla luminescence in each well.

### 2.10. Western Blotting

Total protein was extracted from the cultured CPMs with RIPA lysis buffer (So-larbio, Beijing, China) and PMSF proteinase inhibitors (Solarbio, Beijing, China). A 15 min culture on ice was followed by a 5 min centrifugation at 4 °C at 13,000× *g*. The supernatant was collected. The protein was separated by SDS-PAGE and transferred to PVDF membranes (Bio-Rad Lab, CA, USA). The membrane was then incubated with primary and secondary antibodies according to the standard process [20]. The main antibodies used were anti-*MYOD* (18943-1-AP, Proteintech, 1:1000) and anti-GAPDH (GB15002, Servicebio, 1:2000). The secondary antibodies used were Alexa Fluor^®^ 488-labeled goat anti-mouse IgG HRP (GB25301, Servicebio, 1:5000) and horseradish peroxidase-labeled goat anti-rabbit IgG (GB23303, Servicebio, 1:5000).

### 2.11. Statistical Analysis

All data were statistically analyzed using SPSS 26.0 software (IBM, Armonk, NY, USA). Multiple groups were compared by one-way ANOVA and posttest, and two groups were compared by Student’s t-test (* *p* < 0.05, ** *p* < 0.01). The analysis of the F2 population was conducted according to Model I.

## 3. Results

### 3.1. Genetic Conservation Analysis and Protein Interaction of the QPCTL Gene

**Figure 1:**
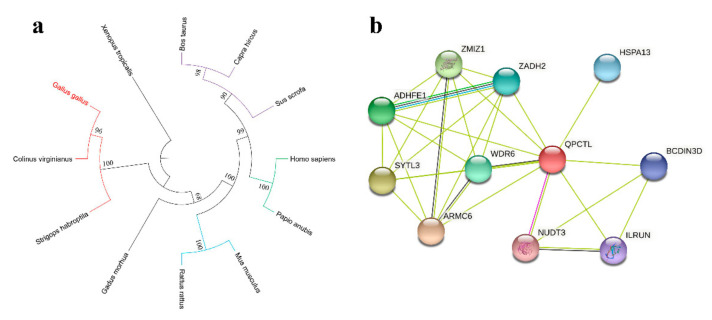
Bioinformatics prediction of *QPCTL*. (**a**) Phylogenetic tree of chicken *QPCTL*. Gallus gallus (chicken), Colinus virginianus (northern bobwhite), Strigops habroptila (kakapo), Bos taurus (cattle), Capra hircus (goat), Sus scrofa (pig), Homo sapiens (human), Papio anubis (olive baboon), Mus musculus (house mouse), Rattus rattus (black rat), and Xenopus tropicalis (tropical clawed frog). (**b**) Interacting proteins of chicken *QPCTL*. The purple line represents the interaction that has been verified by experiments, the black line represents the co-expression relationship, the yellow line represents text mining, and the blue line represents the gene neighborhood.

Phylogenetic trees were constructed by comparing the *QPCTL* amino acid sequences of different species. The *QPCTL* of different species clustered into three large branches. The *QPCTL* of Gallus gallus, Colinus virginianus and Strigops habroptila were clustered in the same large branch, and the genetic distance was small, indicating a close evolutionary relationship between them. Bos taurus, Capra hircus, Sus scrofa, Homo sapiens, Papio anubis, Mus musculus, and Rattus rattus clustered in another large branch. A small genetic distance was detected between these species. The *QPCTL* of different species may be homologous. However, the *QPCTL* of Xenopus tropicalis belongs to a separate branch, which may be the same gene family as other species (Figure 1a). The *QPCTL* interacting protein of the chicken was predicted using the online website String and was found to be related to 10 proteins, including NUDT3 (Figure 1b). These results indicate that *QPCTL* has good sequence conservation in vertebrates and has multiple interacting proteins related to *QPCTL*.

### 3.2. Spatiotemporal Expression of the QPCTL Gene

**Figure 2:**
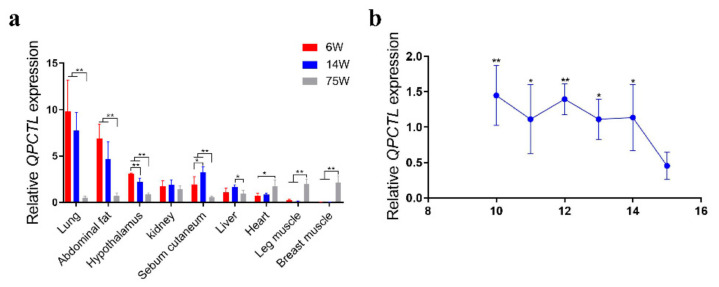
Relative expression levels of the *QPCTL* gene. (**a**) Relative expression patterns of *QPCTL* in different tissues and at different weeks of age in Gushi chickens (*n* = 12). (**b**) Expression of the *QPCTL* gene in breast muscle at different embryonic stages in Xinghua chickens (*n* = 18). * *p* < 0.05; ** *p* < 0.01.

The expression of *QPCTL* in different tissues and at different stages of GS chickens was measured by qPCR. The expression of *QPCTL* in lung, abdominal fat, hypothalamus and sebum tissues of chickens at weeks 6 and 14 was significantly higher than that at week 75 (*p* < 0.01), but the expression of *QPCTL* in leg muscle and breast muscle tissues of chickens at week 75 was significantly higher than that at weeks 6 and 14 (*p* < 0.01). The expression of *QPCTL* in the liver at week 14 was significantly higher than that at week 75 (*p* < 0.05), and the expression in the heart at 6 weeks was significantly lower than that at week 75 (*p* < 0.05) (Figure 2a). Compared with the E15, the expression of *QPCTL* in breast muscle at E10 and E12 was significantly higher than that at E15 (*p* < 0.01), and the expression of *QPCTL* breast muscle at E11, E13 and E14 was significantly higher than that at E15 (*p* < 0.05). Furthermore, *QPCTL* expression in breast muscle showed a trend of a gradual decline with progressing embryonic stages. (Figure 2b). These results suggest that *QPCTL* may be involved in chicken muscle development.

### 3.3. Association between the QPCTL Gene 52/224-bp Multiallelic Indel and ADG of Chickens

We used the SPSS software to analyze the correlation between genotypes and the ADG of chickens; the six genotypes were significantly related to the ADG of chickens at 0–4 weeks and 4–8 weeks (*p* < 0.05), but had no significant correlation with the ADG of the chickens at 8–12 weeks (Table 1). Notably, the AB genotype had a significantly higher ADG than the AC genotype, at 0–4 weeks and 4–8 weeks (*p* = 0.004; *p* = 0.005), whereas the AA genotype exhibited a significantly higher ADG than the AC genotype at 4–8 weeks (*p* = 0.026). Additionally, the AA, BB and AB genotypes had greater ADG, whereas the CC, BC and AC genotypes had a smaller ADG. These results indicated that Indels in the *QPCTL* gene were significantly correlated with the ADG in chickens.

### 3.4. Genotype Frequencies in Different Varieties

We analyzed the frequencies of the different genotypes using GraphPad Prism software. The statistical analysis of six different genotypes showed that the AB and BB genotypes exhibited the highest frequency in white feathered broilers, whereas the frequency of the AC and AA genotypes was low, and the CC genotype was not found. In yellow feathered broilers, the CC and AC genotypes exhibited the highest frequency, whereas the frequency of the AA, AB and BC genotypes was low, and the BB genotype was not found. Six genotypes were distributed in the dual-purpose chickens (Figure S1). These results indicated that different genotypes of *QPCTL* may be affected by artificial breeding.

### 3.5. Relative Expression of QPCTL in Different Genotypes

**Figure 3:**
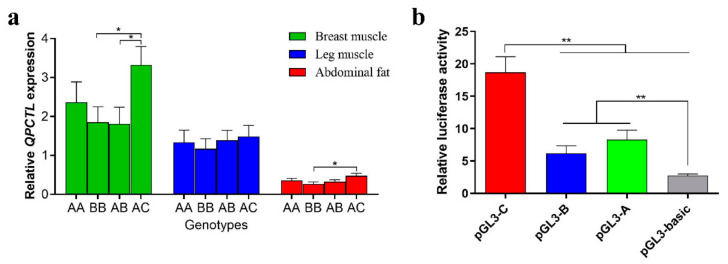
Effects of different Indels on *QPCTL*. (**a**) Expression of the *QPCTL* gene in breast muscle, leg muscle and abdominal fat of Gushi chickens with different genotypes (*n* = 12). We did not collect sufficient tissues from CC and BC genotype individuals to compare the expression of all genotypes. (**b**) Luciferase activity in DF-1 cells transfected with recombinant plasmids. * *p* < 0.05; ** *p* < 0.01.

The expression of *QPCTL* in leg muscle, breast muscle and abdominal fat tissues of different genotypes was measured by qPCR. The expression of the AC genotype in breast muscle tissues was significantly higher than that found in the BB and AB genotypes (*p* < 0.05). The expression of the AC genotype was significantly higher than that of the BB genotype in abdominal adipose tissue (*p* < 0.05). No significant difference among the four genotypes in leg muscle tissue was found (Figure 3a). These results showed that different genotypes affect the expression of *QPCTL*.

### 3.6. Promoter Activity of QPCTL

To determine the effect of Indels in different alleles on promoter activity using Dual-Luciferase, we co-transfected pGL3-basic, pGL3-A, pGL3-B and pGL3-C vectors with PRL-TK into DF-1 cells. The luciferase activity of the pGL3-C vector was significantly higher than that of the pGL3-basic, pGL3-A and pGL3-B vectors (*p* < 0.01), and the luciferase activity of the pGL3-A and pGL3-B vectors was significantly higher than that of the pGL3-basic vector (*p* < 0.01) (Figure 3b). These results indicate that Indels of different alleles affect the activity of promoters.

### 3.7. Prediction of TFBSs in QPCTL Indels

**Figure 4:**
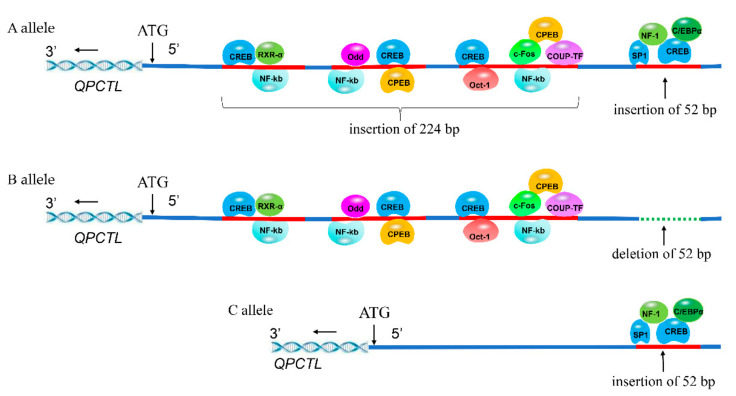
Predicted binding sites of transcription factors in the A, B and C alleles; the A allele (insertions of 52 bp and 224 bp), the B allele (deletion of 52 bp and insertion of 224 bp) and the C allele (insertion of 52 bp). The −362 to −89 (ATG as +1) region existed in three Indels: 112 bp, 96 bp, and 16 bp. In our previous study, we found that these three Indels were simultaneously inserted or deleted in 1806 chickens by genotyping; thus, we regard them as a 224 bp Indel [8].

The transcription factor-binding sites (TFBSs) in the 52/224-bp multiallelic Indels of the *QPCTL* gene promoter were analyzed by Alibaba 2.1. The insertion fragment in the A allele had eleven TFBSs, including CREB, Oct-1, NF-kb, c-Fos, CPEB, COUP-TF, Odd, RXR-α, SP1, NF-1 and C/EBPα; the insertion fragment in the B allele had eight TFBSs, including CREB, Oct-1, NF-kb, c-Fos, CPEB, COUP-TF, Odd and RXR-α; and the insertion fragment in the C allele had four TFBSs, including SP1, NF-1, CREB and C/EBPα. There are 4 CREB, 3 NF-kb and 2 CPEB TFBSs in the A allele insertion fragment, and 3 CREB, 3 NF-kb and 2 CPEB TFBSs in the B allele insertion fragment (Figure 4). These results suggest that the specific transcription factors in the A and B alleles may affect promoter activity.

### 3.8. Effect of the Dietary Nutrition Level on QPCTL Expression

**Figure 5:**
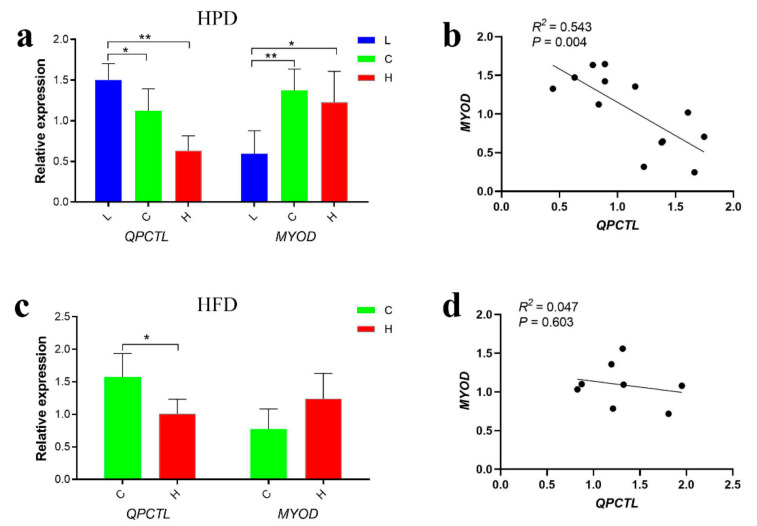
Trend analysis of *QPCTL* and *MYOD* gene expression. (**a**) Expression of *QPCTL* and *MYOD* genes in breast muscle of Gushi chickens fed a high-protein diet (*n* = 18). (**b**) Correlation analysis of *QPCTL* and *MYOD* gene expression in chickens fed a high-protein diet. (**c**) Expression of *QPCTL* and *MYOD* genes in breast muscle of Gushi chickens fed a high-fat diet (*n* = 10). (**d**) Correlation analysis of *QPCTL* and *MYOD* gene expression in chickens fed a high-fat diet. * *p* < 0.05; ** *p* < 0.01.

To investigate the effect of different nutritional levels on the expression of *QPCTL* in breast muscle tissues by qPCR, chickens were fed a high-protein diet (HPD) or a high-fat diet (HFD) for three weeks. The results revealed that the expression of *QPCTL* in the low protein group (L) was significantly higher than that in the control group (C) and high protein group (H) (*p* < 0.05; *p* < 0.01), whereas the expression of *MYOD* in the L group was significantly lower than that in the control group and H group (*p* < 0.05; *p* < 0.01) (Figure 5a). The analysis of breast muscle tissues of HFD-fed chickens showed that the expression of *QPCTL* in the control group (C) was significantly higher than that in the high-fat group (H) (*p* < 0.05); in addition, the expression of *MYOD* in the H group was higher than that in the control group, but this difference was not significant (Figure 5c). The expression levels of *QPCTL* and *MYOD* were significantly negatively correlated in the breast muscle tissues of the HFD (*p* < 0.01) and HPD groups (*p* > 0.05) (Figure 5b,d). These results suggest that *QPCTL* and *MYOD* may have opposite functions in chicken muscle development.

### 3.9. QPCTL Promotes the Proliferation of CPMs

**Figure 6:**
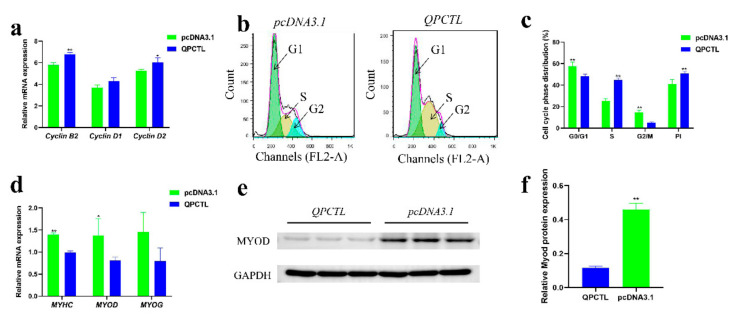
*QPCTL* promotes the proliferation of CPMs and inhibits their differentiation. (**a**) Expression of cell cycle-related genes after the overexpression of *QPCTL*. (**b**,**c**) Cell cycle analysis of *QPCTL* overexpression in CPMs. Count represents the number of cells counted; FL2-A is the propidium iodide fluorescence area signal, representing the DNA content of cells. The result of (**c**) comes from (**b**). (**d**) Expression of myoblast differentiation-related genes after the overexpression of *QPCTL*. (**e**,**f**) Protein level of *MYOD* in differentiated myoblasts transfected with the *QPCTL* overexpression vector. * *p* < 0.05; ** *p* < 0.01.

To understand the function of *QPCTL*, we conducted an overexpression experiment to evaluate its role in cell proliferation. We measured by qPCR the mRNA expression of cell cycle-related genes, such as cyclin B2, cyclin D1 and cyclin D2, in CPMs. The results suggested that the cyclin B2 and cyclin D2 genes were significantly upregulated after transfection of the *QPCTL* overexpression vector (*p* < 0.01; *p* < 0.05), and cyclin D1 showed an upward trend, but the difference was not significant (Figure 6a). Flow cytometry was then performed to detect the cell cycle status. The overexpression of *QPCTL* significantly reduced the number of cells staying in G0/G1 and G2/M phases, significantly increased the number of cells at the S phase, and significantly increased the proliferation activity (PI) of cells (*p* < 0.01) (Figure 6b,c). The above results indicate that the overexpression of *QPCTL* promotes the proliferation of primary myoblasts.

### 3.10. QPCTL Inhibits the Differentiation of CPMs

To further study the potential role of *QPCTL*, CPMs were induced to differentiate for two days in vitro, and the mRNA levels of *MYHC*, *MYOD*, and *MYOG* genes related to myoblast differentiation were measured by qPCR [21]. Moreover, the protein level of MYOD after differentiation of CPMs was measured by Western blot. Compared with that in the control group, the mRNA expression of *MYHC* and *MYOD* in cells transfected with the *QPCTL* overexpression vector was significantly decreased (*p* < 0.01; *p* < 0.05) (Figure 6d). The protein level of MYOD in CPMs after the overexpression of *QPCTL* was significantly decreased (*p* < 0.01) (Figure 6e,f). These results indicate that *QPCTL* inhibits the differentiation of CPMs.

## 4. Discussion

The chicken weight is a heritable trait, with a heritability of 0.24–0.47% [22]. In addition, chicken growth and carcass performance are the main breeding indicators [23]. The growth rate and weight of native Chinese chickens are relatively low compared with those of commercial broilers [24]. Recent studies have found that, in humans, the interacting protein NUDT3 of *QPCTL* is an important genetic biomarker of sarcopenia in elderly individuals. The locus of this gene is related to lipid and energy metabolism [25]. Therefore, we studied the correlation between the growth rate of F2 populations and multiallelic Indels of the *QPCTL* gene, and found that the multiallelic Indels were significantly correlated with the ADG at the two stages (Table 1). At the early growth stage of chickens, the ADG of AA and AB genotype chickens was significantly higher than that of AC genotype chickens. AA and AB genotype chickens may have a faster growth rate during the development process, whereas AC genotype chickens may have a slower growth rate. BC genotype has the lowest ADG in three periods; however, there was no significant difference between BC and other genotypes, which may be due to the number of individuals with BC genotype being relatively small, and the weight between individuals may have a large standard error (SE). From weeks 0 to 12, the F2 population is all in the growth period. The weight of chickens increases with the increase of their age. Genetic factors, diseases and feed conversion rate may affect the growth of chickens [26,27,28]. We speculate that the increase of the SE over time may be related to the weight gain of chickens. Population size and more genotypes may also be the reason for high SE, but the number of 804 F2 population far exceeded some previously published studies [29]. The high SE in the last period is probably the reason for the insignificant results. In addition, the frequency of the AB and BB genotypes was the highest in white feathered broilers, whereas the frequency of the AC genotype was relatively low, and the CC genotype was not found. In yellow feathered broilers, the CC and AC genotypes exhibited the highest frequency, and the BB genotype was not found. The frequency of dominant genotypes related to growth and development may have increased during the process of high-intensity artificial breeding of white feathered broilers [30]. In our previous study, the AA, BB and AB genotypes had larger weight and carcass traits, whereas the AC genotype was the inferior genotype with respect to weight and carcass traits [8]. In conclusion, the AC genotype is the inferior genotype based on chicken growth traits, and multiallelic Indels in the promoter region of *QPCTL* are related to the growth and development of chickens.

To explore why individuals with different genotypes have different growth rates, we then analyzed the expression of different genotypes in abdominal fat, leg muscle and breast muscle. The results showed that the expression of the AC genotype in breast muscle was significantly higher than that of the AB and BB genotypes (Figure 3a). Interestingly, the ADG of the AC genotype was significantly lower than that of the AB and AA genotypes. Based on the transcriptional activity results, the pGL3-C vector produced significantly more luciferase activity than pGL3-A and pGL3-B. (Figure 3b). This result suggests that insertions in the promoter region of *QPCTL* may be related to the binding of inhibitory transcription factors, which may reduce the activity of *QPCTL* and have beneficial effects on the growth and development of chickens. These results suggest that the expression of *QPCTL* may be negatively correlated with chicken growth, and that different Indels affect the expression of *QPCTL*.

In recent decades, a growing body of evidence has suggested that the regulation of gene expression in eukaryotes is a complex regulatory process involving many factors, including promoters, introns, and 5′ and 3′ untranslated regions in specific genes [26,31]. Transcription factors play key roles in controlling gene expression. We predicted the transcription factor binding sites of insertion fragments of A, B and C alleles. The results indicated the presence of Oct-1 transcription factor binding sites in the insertion fragments of the A and B alleles (Figure 4). Previous studies have shown that Oct-1 is an important transcription inhibitor of the *p15INK4b* gene, which may participate in the regulation of cell aging by inhibiting gene transcription [32]. An analysis of the expression of different genotypes in different tissues and a dual-luciferase experiment of *QPCTL* indicate that Oct-1, a potential transcription factor in A and B alleles, may inhibit gene expression by blocking the activity of the *QPCTL* promoter, which leads to significant differences in the growth rate of individuals with different genotypes in F2 resource populations. In our previous studies, we also found that intron binding to inhibitory transcription factors affect gene expression [16].

Compared with the A and B alleles, the C allele has higher transcriptional activity. The qPCR results of different genotypes in breast muscle tissue showed that the expression of *QPCTL* in individuals with the AC genotype was significantly higher than that in individuals with the AB and BB genotypes. Compared with the AA genotype, the expression of *QPCTL* in the AC genotype tended to increase, but the difference was not significant. Unfortunately, we did not collect sufficient tissues from CC and BC genotype individuals to compare the expression of all genotypes. In addition, the 0–4 ADG and 4–8 ADG of the AC genotype were significantly lower than those of the AB and AA genotypes. In conclusion, high expression of *QPCTL* in individuals with the AC genotype may be the reason for their relatively low ADG.

The analysis of the temporal and spatial expression of *QPCTL* showed that the expression of the *QPCTL* gene in the leg muscle and breast muscle of 75-week-old Gushi chickens increased significantly with increasing age, whereas the expression of the *QPCTL* gene in the abdominal fat of chickens decreased significantly (Figure 2a). It is worth noting that 6 weeks and 14 weeks are the periods of rapid growth of local chickens, and *QPCTL* is only expressed at low levels at 6 weeks and 14 weeks during the growth period of native chickens [33]. In addition, with increasing embryonic age, the expression of *QPCTL* in chicken breast muscle gradually decreased (Figure 2b). The results obtained with the HFD and HPD indicated that the expression levels of *QPCTL* and *MYOD* in breast muscle tissues were negatively correlated (Figure 5). These results suggest that *QPCTL* may be involved in chicken muscle development and that *QPCTL* may inhibit muscle development. Recent studies have shown that the knockout of *TMEM182* in mice can increase the size of muscle fibers, whereas the overexpression of *TMEM182* can cause muscle atrophy in chickens [34]. This gene is a novel negative regulator of muscle regeneration and myogenic differentiation, and *QPCTL* may have a similar function.

To conduct experiments in vitro, we synthesized the *QPCTL* overexpression vector. The results from a series of experiments showed that the overexpression of *QPCTL* significantly increased the number of cells at the S phase, promoted the proliferation of CPMs, and upregulated the expression of cyclin B2 and cyclin D2 genes related to cell proliferation. In addition, the overexpression of *QPCTL* inhibited the expression of *MYHC* and *MYOD*, which are markers of CPM differentiation, and reduced the protein expression level of MYOD (Figure 6). Similar studies have previously reported that chicken *c-Myc* can promote CPM proliferation and inhibit CPM differentiation, and this gene participates in the regulation of CPM proliferation and differentiation through targeted cell cycle pathways [35]. These results suggest that *QPCTL* may participate in chicken muscle development by promoting CPM proliferation and inhibiting CPM differentiation, but the detailed molecular mechanism still needs further study.

## 5. Conclusions

In short, the results from our association analysis indicated that 52/224-bp multiallelic Indels in the promoter region of *QPCTL* could significantly affect the ADG of chickens, and the AC genotype was the inferior genotype based on chicken growth traits. Insertion fragments in A and B alleles can reduce promoter activity and inhibit the expression of *QPCTL*. The analysis of different dietary supplements indicated that the expression of *QPCTL* is negatively correlated with the expression of *MYOD*. Through further research, we found that *QPCTL* can promote the proliferation of myoblasts and inhibit the differentiation of myoblasts. Therefore, individuals with the AA, BB and AB genotypes have lower gene expression but higher growth rates. In conclusion, the results of this study suggest that Indels in the *QPCTL* promoter region can be used as molecular markers for poultry molecular breeding. The *QPCTL* gene may participate in chicken embryonic development and early growth and can serve as a candidate gene for chicken growth and development.

## Figures and Tables

**Table 1:** Association analysis of the *QPCTL* multiallelic Indels with growth traits in the Gushi x Anka F2 population.

Traits	Mean ± SE						*p*-Value
	AA	BB	CC	AB	AC	BC	
0–4 ADG	10.44 ± 0.10 ^ab^	10.75 ± 0.31 ^ab^	10.23 ± 0.32 ^ab^	10.66 ± 0.13 ^a^	10.17 ± 0.11 ^b^	9.89 ± 0.79 ^ab^	0.045
4–8 ADG	18.7 ± 0.31 ^a^	18.58 ± 0.77 ^ab^	17.53 ± 0.66 ^ab^	19.11 ± 0.42 ^a^	17.69 ± 0.30^b^	17.03 ± 1.58 ^ab^	0.048
8–12 ADG	21.25 ± 0.51	22.28 ± 1.85	20.02 ± 1.18	21.43 ± 0.63	20.44 ± 0.57	19.4 ± 2.14	0.645

Note: SE = standard error of the mean; 0 to 4, 4 to 8 and 8 to 12 ADG = daily gains at 0 to 4, 4 to 8 and 8 to 12 weeks (g/day), respectively. Means with different superscripts indicate significant differences (different lowercase letters indicate *p* < 0.05; the same letters indicate *p* > 0.05). AA genotype (*n* = 287), BB genotype (*n* = 31), CC genotype (*n* = 42), AB genotype (*n* = 188), AC genotype (*n* = 243), BC genotype (*n* = 13).

## Data Availability

All data in the current study can be obtained from the corresponding author upon reasonable request.

## Supplementary Materials

The following supporting information can be downloaded at: https://www.mdpi.com/article/10.3390/ani12243535/s1, Figure S1: (a) Electrophoresis (2.5%) patterns showing the amplification results for *QPCTL* gene. (b) Percentage of different genotypes in the different populations; Table S1: Details of primer pairs.
