# Supplementary material for: QPCTL Affects the Daily Weight Gain of the F2 Population and Regulates Myogenic Cell Proliferation and Differentiation in Chickens
Source: Animals (Basel). 2022 Dec 14;12(24):3535. doi: 10.3390/ani12243535 (PMC9774964; doi:10.3390/ani12243535)
Supplement: Supplementary file 1 [file animals-12-03535-s001.zip › animals-2065340-supplementary.pdf]

QPCTL Affects the Daily Weight Gain of the F2 Population and Regulates Myogenic Cell Proliferation and Differentiation in Chickens

Tuanhui Ren[1,2,3], Xiuxian Yang[1,2], Wujian Lin[1,2], Zihao Zhang[1,2,] Shizi He[1,2], Wangyu Li[1,2], Zhuanjian Li[3*], Xiquan Zhang[1,2*]

1.Department of Animal Genetics, Breeding and Reproduction, College of Animal Science, South China Agricultural University, Guangzhou 510642, China;

2.Guangdong Provincial Key Lab of Agro-Animal Genomics and Molecular Breeding, Key Laboratory of Chicken Genetics Breeding and Reproduction, Ministry of Agriculture, Guangzhou 510642, China;

3.College of Animal Science and Technology, Henan Agricultural University, Zhengzhou 450046, China.

*** Correspondence:** lizhuanjian@163.com (Z.L.); xqzhang@scau.edu.cn (X.Z.)

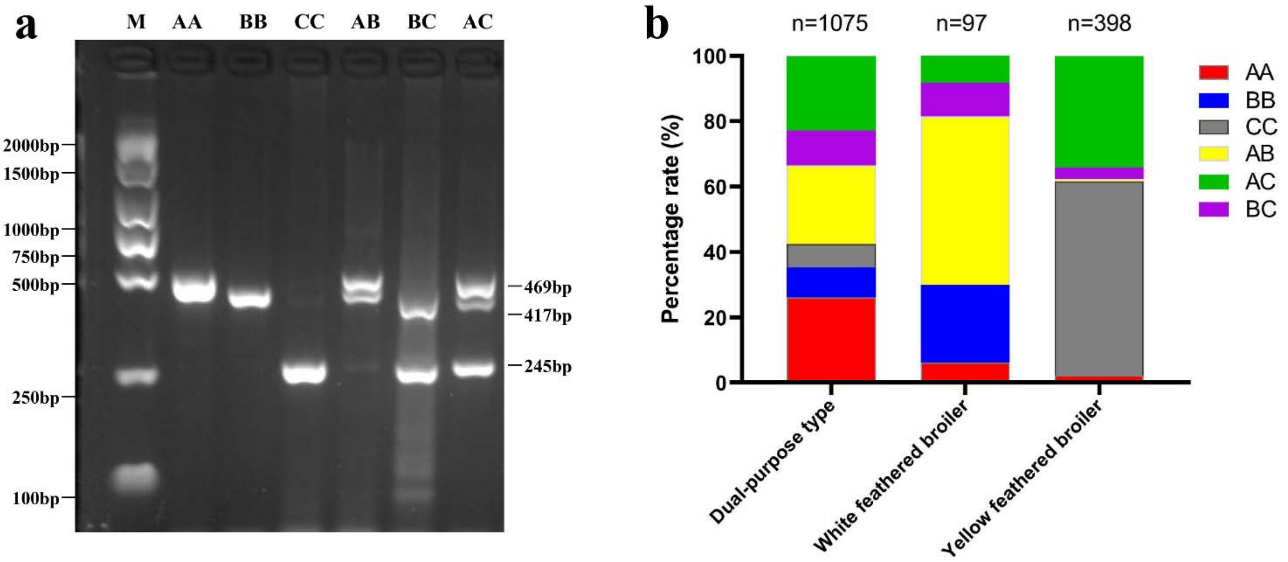

Figure S1. (a) Electrophoresis (2.5%) patterns showing the amplification results for *QPCTL* gene. AA, BB, CC, AB, AC and BC are the six different genotypes, and M represents BM2000. (b) Percentage of different genotypes in the different populations. Dual-purpose type (Gushi (female, *n* = 143), Lushi (female, *n* = 192), Changshun (female, *n* = 135), Xichuan (female, *n* = 323), A line (female, *n* = 29), D line (female, *n* = 30), H line (female, *n* = 26), M line (female, *n* = 30), 103 line dwarf chicken (female, *n* = 48), Xinghua dwarf chicken (female, *n* = 48) and Yellow dwarf chicken (female, *n* = 71)), white feathered broilers (Recessive White Rock chicken (male, *n* = 57) and Arbor Acres chicken (male, *n* = 40)), and yellow feathered broilers (Ma-Huang chicken (male, *n* = 398)). A line, D line, H line and M line all from Gushi chicken hybrid lines, 103 line dwarf chicken, Xinghua dwarf chicken and Yellow dwarf chicken all from dwarf chicken hybrid lines.

Table S1. Details of primer pairs.

Gene name	Primer sequences (5'-3')	Sizes (bp)
QPCTL	F: GCGTGTCACTACGACACCAA<br>R: CTTCTCCATCCAGGAACAGC	193
Cyclin D1	F: CAGAAGTGCGAAGAGGAAGT<br>R: CTGATGGAGTTGTCGGTGTA	188
Cyclin D2	F: AACTTGCTCTACGACGACC<br>R: TTCACAGACCTCCAACATC	150
Cyclin B2	F: CAGTAAAGGCTACGAAAG<br>R: ACATCCATAGGGACAGG	133
MYHC	F: CTCCTCACGCTTTGGTAA<br>R: TGATAGTCGTATGGGTTGGT	213
MYOD	F: GCTACTACACGGAATCACCAAAT<br>R: CTGGGCTCCACTGTCACTCA	200
MYOG	F: CGGAGGCTGAAGAAGGTGAA<br>R: CGGTCCTCTGCCTGGTCAT	320
β-actin	F: GACTGACCGCGTTACTCCCA<br>R: CCAACCATCACACCCTGATGTC	166
QPCTL-Genotyping	F: GACTGACCGCGTTACTCCCA<br>R: CCAACCATCACACCCTGATGTC	245, 417, 469

Note: Tm values of all primers were 60 °C; The primers of *QPCTL*, *Cyclin D1*, *Cyclin D2*, *Cyclin B2*, *MYHC*, *MYOD*, *MYOG* and *β-actin* were used for qRT-PCR, and the primers of *QPCTL*-Genotyping were used for Indels genotyping.
